# Unusual cutaneous manifestations of disseminated *Cryptococcus gattii* infection in an immunocompetent host

**DOI:** 10.1590/S1678-9946202567078

**Published:** 2025-11-03

**Authors:** Carolina Andrade Lopes, Stephanie Victoria Camargo Leão Edelmuth, Alessandra Luna-Muschi, Mariane Taborda, Vítor Falcão de Oliveira, Adriana Satie Gonçalves Kono Magri, Afonso Rafael da Silva, Ana Catharina de Seixas Santos Nastri, Marcello Mihailenko Chaves Magri

**Affiliations:** 1 Universidade de São Paulo, Faculdade de Medicina, Hospital das Clínicas, Divisão de Clínica de Moléstias Infecciosas e Parasitárias, São Paulo, São Paulo, Brazil; 2 Universidade de São Paulo, Faculdade de Medicina, Hospital das Clínicas, Divisão de Laboratório Central, São Paulo, São Paulo, Brazil

**Keywords:** Cryptococcus gattii, Disseminated cryptococcosis, Cutaneous cryptococcosis

## Abstract

We report a rare case of disseminated *Cryptococcus gattii* species complex (CGSC) infection in a 51-year-old immunocompetent man who initially had extensive ulcerated skin nodules, an uncommon cutaneous manifestation of cryptococcosis. Positron emission tomography/computed tomography (PET/CT) revealed hypermetabolic lesions in the skin and lungs, while brain magnetic resonance imaging (MRI) demonstrated multiple cryptococcomas with mass effect. The diagnosis was confirmed by skin and lung biopsies and a positive serum cryptococcal antigen test. Induction therapy with lipid complex amphotericin B, flucytosine, and corticosteroids was initiated. This case highlights the importance of recognizing atypical cutaneous lesions as potential indicators of disseminated CGSC infection in immunocompetent individuals, and underscores the need for early antifungal management.

## INTRODUCTION

*Cryptococcus gattii* species complex (CGSC) comprises encapsulated yeasts capable of causing severe systemic infections, particularly in immunocompetent individuals. Once restricted to tropical and subtropical regions, CGSC has emerged in temperate climates, with outbreaks reported in Canada and Australia. Infections most commonly affect the lungs or central nervous system (CNS), whereas cutaneous lesions are rare and usually indicate dissemination^1^. CGSC is listed as a high-priority fungal pathogen by the World Health Organization, due to its potential to cause a life-threatening disease and its limited access to diagnosis and treatment in many regions^2-6^.

Unlike *C. neoformans* species complex (CNSC), CGSC often induces exuberant inflammatory responses and forms cryptococcomas, leading to a more chronic, mass-like presentation in the lungs or central nervous system (CNS)^1-7^. The recently published global guideline for cryptococcosis management emphasizes that CGSC disproportionately affects non-HIV, non-transplant hosts, and typically requires longer induction therapy in CNS disease^7^.

In Brazil, CGSC accounts for a significant proportion of cryptococcosis in HIV-negative patients, especially in the North and Northeast, where genotype VGII predominates. Diagnosing these cases can be difficult due to nonspecific symptoms and low clinical suspicion in the absence of overt immunosuppression^2^. We describe a case of disseminated CGSC infection in an immunocompetent Brazilian man with ulcerated skin lesions, a pulmonary mass, and CNS involvement. The cutaneous findings were initially misleading and may mimic other endemic mycoses or neoplastic conditions, contributing to diagnostic delay.

### Ethics

Written informed consent was obtained from the patient for the publication of this case report and accompanying images. Ethical approval for the study was obtained from the local ethics committee (protocol N° 72638123.1.0000.0068).

## CASE REPORT

A 51-year-old man, a baker residing in the metropolitan area of Sao Paulo, Brazil, with a medical history of hypertension, type 2 diabetes mellitus, dyslipidemia, and class II obesity (BMI: 37.4), had a two-month history of progressively enlarging nodule-tumoral skin lesions. The lesions initially appeared on the scalp and later involved the face and upper limbs; they were friable nodule-tumoral lesions with central ulceration and hemorrhagic crusts. The patient also reported daily holocranial headaches, nausea, vomiting, dry cough, weight loss of 30 kg, and intermittent fevers up to 39 °C.

Initial treatment at a primary care unit with cephalexin and dipyrone for presumed skin infection was ineffective. A new nodule-tumoral lesion appeared on the anterior chest one month later, and 15 days after, he developed dysphonia.

**Figure 1:**
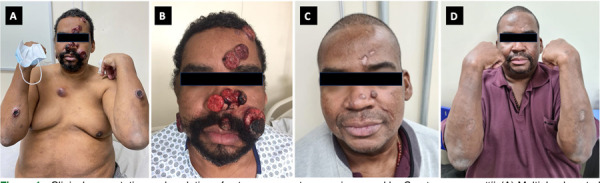
Clinical presentation and evolution of cutaneous cryptococcosis caused by *Cryptococcus gattii*: (A) Multiple ulcerated nodule-tumoral lesions on the face, chest, and upper limbs; (B) Extensive facial nodule-tumoral lesions on the first day of hospitalization; (C) Partial regression of cutaneous lesions six months after initiation of antifungal therapy; (D) Residual scarring and regression of lesions one year after treatment.

Physical examination revealed an afebrile, well-appearing patient, alert and oriented, without meningeal signs. He had a large (7 × 7 cm), firm, and erythematous-violaceous nodule-tumoral on the anterior thorax, and multiple ulcerated nodule-tumoral lesions with central necrosis on the scalp, face, and upper limbs (Figures 1A and 1B). Neurological examination showed preserved muscle strength and reflexes, without focal deficits.

**Figure 2:**
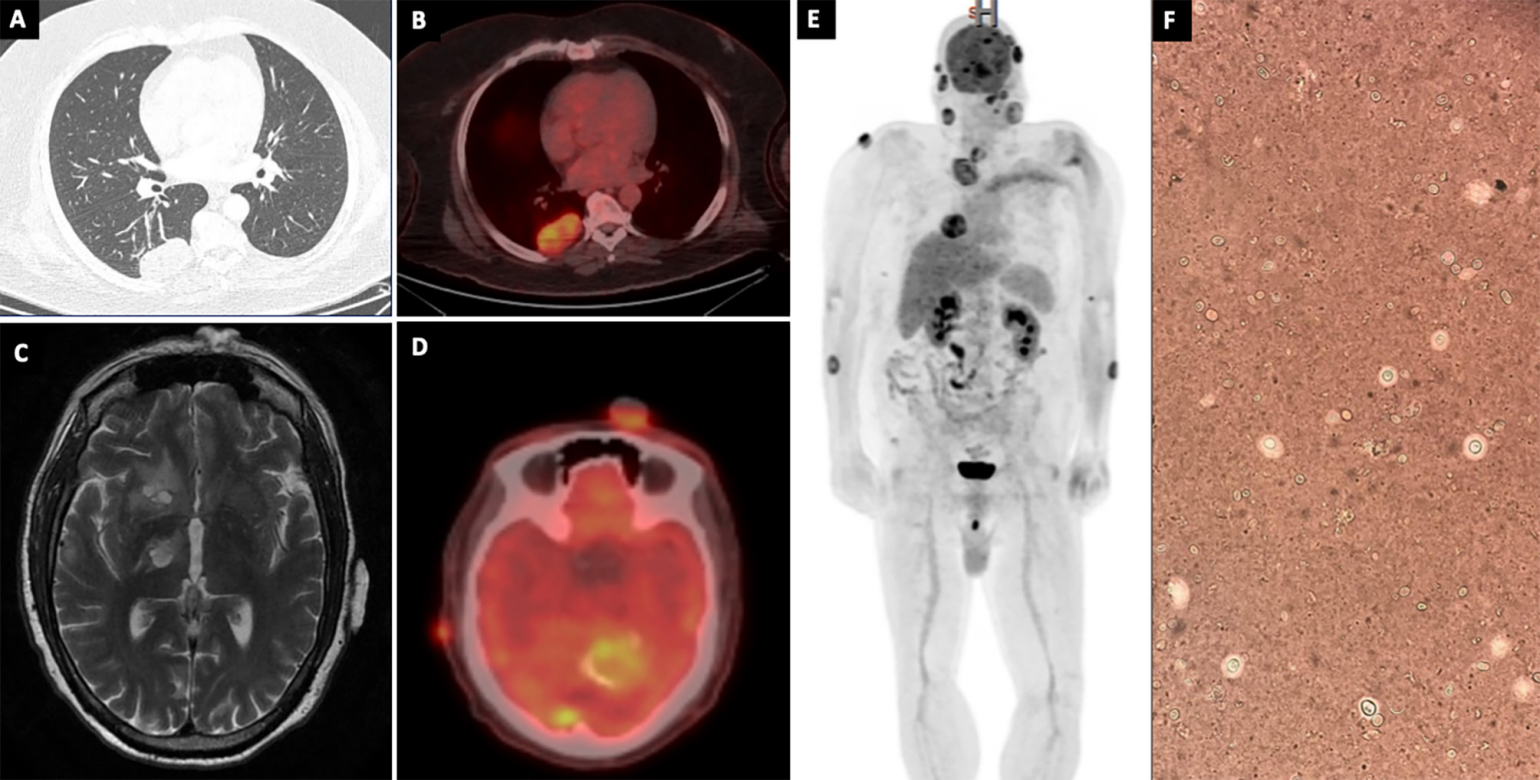
Radiological examinations showing the lesions of the patient with *Cryptococcus gattii*: (A) Homogeneous, well-defined expansile mass with a broad pleural base, located in the periphery of the superior segment of the right lower lobe, extending from the 5th to the 7th costal arches, with which it is in close contact. It measures 5.3 × 3.1 × 4.3 cm; (B) PET scan showing a homogeneous expansile pulmonary mass in the periphery of the superior segment of the right lower lobe; (C) Brain T2 MRI demonstrated multiple intra-axial lesions in both infra- and supratentorial compartments, with a large lesion in the posterior fossa; (D) PET scan showing a solid expansile lesion with calcification foci in the left cerebellum. Multiple scattered foci in the cerebral parenchyma; (E) Multiple nodular formations in the cutaneous/subcutaneous planes of the face, thoracic and abdominal wall, and upper limbs; (F) India ink staining showing yeast-like cells suggestive of *Cryptococcus* spp.

Figure 2 shows the main imaging findings. Initial imaging showed a homogeneous pulmonary mass with regular margins in the right lower lobe, measuring 5.3 × 3.1 × 4.3 cm. Neck CT revealed nodular lesions in subcutaneous facial tissue and signs of right vocal cord paralysis. Brain MRI demonstrated multiple intra-axial lesions in both infra- and supratentorial compartments, with a large lesion in the posterior fossa. Whole-body 18F-FDG PET/CT revealed hypermetabolic activity in the pulmonary consolidations and cutaneous lesions, consistent with active granulomatous inflammation and disseminated fungal infection. Lumbar puncture was initially contraindicated due to the risk of brain herniation.

Skin biopsy revealed encapsulated yeasts consistent with *Cryptococcus* spp. (Figure 2F), whereas lung biopsy demonstrated fungal elements with positive Grocott and Mucicarmine staining, and culture confirmed CGSC infection. Serum cryptococcal antigen (CrAg) lateral flow assay was positive. Fungal culture from skin tissue identified CGSC. Blood cultures and PCR for *Leishmania* spp. were negative. T-cell clonality testing and immunophenotyping showed no evidence of lymphoproliferative disorder. HIV, HBV, HCV, HTLV, and syphilis serologies were negative. An immunology consultation was requested to investigate primary immunodeficiencies, and no abnormalities were identified.

Induction therapy was initiated with amphotericin B lipid complex (5 mg/kg/day) and flucytosine (25 mg/kg every 6 h), adjusted for renal function, given the presumptive diagnosis of disseminated CGSC infection with probable CNS involvement. Dexamethasone was added due to CNS mass effect. The patient showed marked clinical improvement following a six-week induction regimen and consolidation therapy with oral fluconazole (Figures 1C and 1D).

## DISCUSSION

This case highlights a rare but clinically important presentation of disseminated CGSC infection in an immunocompetent individual, involving the skin, lungs, and central nervous system. The initial manifestation with striking ulcerated skin nodules, which is an uncommon and often overlooked feature of cryptococcosis, underscores the importance of recognizing cutaneous lesions as potential indicators of systemic fungal dissemination. Cutaneous involvement by CGSC is rarely reported, particularly in hosts without identifiable immunosuppression, and the exuberant morphology observed in our patient provided a valuable diagnostic window in the absence of early neurological symptoms or lumbar puncture. The subacute course and lack of overt immunosuppression led to diagnostic delay—an issue commonly encountered in CGSC disease.

In the MSG07 multinational cohort, Baddley et al. (2021) evaluated 150 patients with CGSC infection and found that they were more likely to be immunocompetent than those with CNSC infection (58.7% vs. 21.3%), to have pulmonary disease (60.7% vs. 32.9%), and to have a longer median duration of symptoms prior to diagnosis (52 vs. 36 days; p=0.01)^8^. Our case aligns with these findings: the patient was immunocompetent, experienced more than eight weeks of symptoms before diagnosis, and had both pulmonary lesions and CNS involvement. Radiologically, the presence of large cerebellar cryptococcomas causing mass effect is also consistent with data from MSG07, in which CNS lesions were more frequent in CGSC cases (37.1% vs. 13.9% for CNSC, p<0.001) and often located in the posterior fossa or brainstem. These mass-like lesions reflect the granulomatous response commonly triggered by CGSC, in contrast to the more diffuse meningeal enhancement seen with CNSC. Additionally, neurosurgical consultation was more frequently required in CGSC cases within the cohort (17.3% vs. 4.4%; p<0.01), mostly due to obstructive hydrocephalus or significant mass effect, clinical features also observed in our patient, leading to a delay in lumbar puncture and the use of corticosteroids to manage perilesional edema.

Although cutaneous involvement is less frequent in MSG07 (8.7% of CGSC cases), it may be an early diagnostic clue and route for biopsy in disseminated disease. In our case, skin ulceration led to histopathological confirmation and facilitated microbiological identification, reinforcing the importance of recognizing cutaneous cryptococcosis as a window to systemic infection^8^.

Two recent large-scale studies have further helped characterize the clinical spectrum and prognostic factors of CGSC infection in HIV-negative individuals. In a retrospective cohort, Galanis *et al*.^9^ analyzed 258 cases reported across Australia, British Columbia, and the US Pacific Northwest. Among 218 hospitalized patients, 66.1% had extrapulmonary disease, primarily involving the CNS, and 33.9% had isolated pulmonary disease. Extrapulmonary disease was more frequent among younger patients, those infected with VGI strains, and residents of Australia. In contrast, isolated pulmonary infection was associated with comorbidities and higher mortality. Independent predictors of death included age ≥70 years, chronic lung disease, and immunocompromised status. Notably, among the 144 patients with extrapulmonary involvement, only one had a positive culture from soft tissue, with no additional clinical detail, underscoring the rarity of cutaneous dissemination. Despite guideline recommendations, only 40% of patients received at least 14 days of induction therapy with amphotericin B. Our patient received induction therapy with ABLC and 5FC for six weeks, in accordance with guideline recommendations for CNS involvement with cryptococcomas or pseudocysts. Although L-AmB is the preferred formulation due to superior CSF penetration and reduced toxicity, ABLC is considered an acceptable alternative and was the formulation provided by the Brazilian Ministry of Health at the time. Corticosteroids were introduced to manage perilesional edema and radiological evidence of mass effect.

In a separate multicenter study, Coussement et al.^10^ evaluated 475 cases of cryptococcosis across 46 hospitals in Australia and New Zealand. CGSC infection occurred predominantly in HIV-negative patients (94.3%), many of whom had no recognized immunosuppression, although comorbidities such as diabetes or chronic pulmonary disease were frequent. Pulmonary and CNS involvement were common (75.1% and 45.8%, respectively), and disseminated disease was observed in 40.1% of cases. Cutaneous lesions were rare overall but more frequently observed in immunocompromised patients (7.7%) compared to apparently immunocompetent individuals (1.2%). These manifestations included nodules, plaques, or ulcers, although detailed morphological descriptions were not consistently available. These findings support that while cutaneous involvement is uncommon in CGSC disease, it may occur across a spectrum of immune statuses and can serve as a valuable early diagnostic clue in disseminated infection. Ulcerated nodules on the scalp and limbs were the initial clinical manifestation in our patient, and skin biopsy played a pivotal role in establishing the diagnosis.

Diagnosis in such settings often relies on extrapulmonary biopsy, imaging, and serum cryptococcal antigen testing. Although CrAg lateral flow assay has high sensitivity, it is less validated for detecting CNS disease in CGSC compared to CNSC^7,11^. Histopathology and culture from skin and lung specimens were critical in our case, supporting current recommendations for aggressive tissue-based diagnosis when CNS access is restricted^7^. Coussement *et al*.^10^ found that serum cryptococcal antigen was positive in 97.3% of CGSC infections, and high titers were strongly associated with CNS involvement. Compared to CNSC, CGSC was more frequently associated with cerebral cryptococcomas (45.2% vs. 12%) and less commonly with fungemia (<2%). One-year mortality was lowest among HIV-negative individuals with CGSC infection (11.1%).

Recent studies have elucidated distinct aspects of CGSC pathogenesis compared to CNSC. Although both species share classical virulence factors, such as a polysaccharide capsule, melanin, urease, titan cell formation, and the ability to grow at 37 °C, CGSC shows unique features that enhance its pathogenicity in immunocompetent hosts. The capsule of CGSC differs structurally and functionally from that of CNSC, displaying lower immunogenicity and greater capacity to evade phagocytosis. Titan cells are more frequently observed in CGSC strains, particularly those of VGI and VGII genotypes, and contribute to phagocytic resistance and pulmonary persistence. Moreover, CGSC modulates macrophage polarization toward an M2 phenotype, impairs dendritic cell maturation, and dampens Th1 and Th17 immune responses, which may explain its ability to cause disease in the absence of overt immunosuppression. Several conserved fungal signaling pathways, including the cyclic adenosine monophosphate/protein kinase A (cAMP–PKA) pathway and the high-osmolarity glycerol/mitogen-activated protein kinase (HOG–MAPK) pathway, regulate the expression of these virulence traits and promote adaptation to intracellular survival and host-derived stress conditions^1,12-14^.

Primary cutaneous cryptococcosis (PCC) is an increasingly recognized clinical entity, characterized by localized skin infection without evidence of systemic dissemination. PCC has been described in both immunocompromised and immunocompetent hosts, often presenting as ulcerated or tumor-like lesions at sites of skin trauma or inoculation. Importantly, CGSC has also been reported as a causative agent, broadening the spectrum of species involved. Recognizing PCC is relevant because it differs from disseminated cutaneous cryptococcosis in terms of pathogenesis, clinical course, and therapeutic implications. Recent reviews emphasize its emerging importance and provide updated concepts and management strategies^15^.

This case underscores the importance of considering CGSC in the differential diagnosis of cutaneous and pulmonary lesions, especially in endemic areas. A high index of suspicion, aggressive diagnostic workup, and individualized antifungal management are crucial to improving outcomes in immunocompetent hosts with disseminated cryptococcosis.

## Data Availability

The anonymized dataset generated during this study is available from the corresponding author upon reasonable request.
